# Dilated Cardiomyopathy in a Young Patient With an *FLNC* Gene Mutation

**DOI:** 10.1155/carm/7792307

**Published:** 2025-06-08

**Authors:** Alex David Sotomayor-Julio, Andrea Facio-Lince García, Wikler Bernal-Torres, Sebastián Seni-Molina, Juan David López-Ponce de León

**Affiliations:** ^1^Cardiology Service, Fundación Valle del Lili, Cali, Colombia; ^2^Faculty of Health Sciences, Universidad Icesi, Cali, Colombia; ^3^Clinical Research Center, Fundación Valle del Lili, Cali, Colombia

**Keywords:** case report, dilated cardiomyopathy, *FLNC* mutation, genetic testing, heart failure

## Abstract

**Background:** Dilated cardiomyopathy is a leading cause of heart failure and heart transplantation. Among its etiologies, genetic variants account for up to 35% of cases. Variants in the *FLNC* gene have gained recognition due to their association with a higher risk of major ventricular arrhythmias and sudden cardiac death. Early identification and intervention are critical to improving patient outcomes.

**Case Presentation:** We present the case of a 28-year-old male with no cardiovascular history who presented with ischemic stroke. Neurological improvement was noted following thrombolysis. Extensive testing ruled out infectious, thrombotic, and autoimmune causes. Subsequent evaluation revealed severe left ventricular systolic dysfunction (ejection fraction of 20%) and biventricular dilated cardiomyopathy. Genetic testing identified a likely pathogenic *FLNC* variant NM_001458.5(FLNC):c.1156G>T; p.Glu386∗, confirming the diagnosis of *FLNC*-associated dilated cardiomyopathy.

**Discussion:** This case highlights the importance of investigating genetic causes in young patients presenting with unexplained dilated cardiomyopathy. Although truncating *FLNC* mutations are rare, they are associated with adverse outcomes, including major ventricular arrhythmias and sudden cardiac death. Atypical biventricular involvement suggests overlapping phenotypes, complicating the diagnostic process. Advanced imaging modalities, comprehensive management strategies, and early genetic testing are crucial to optimizing patient outcomes.

## 1. Background

Cardiomyopathies are myocardial disorders characterized by structural and functional abnormalities of the myocardium, without sufficient underlying causes such as hypertension, coronary artery disease, congenital heart defects, or valvular disease. The latest European Society of Cardiology guideline classifies cardiomyopathies into several phenotypes: hypertrophic cardiomyopathy (HCM), dilated cardiomyopathy (DCM), nondilated left ventricular cardiomyopathy (NDLVC), arrhythmogenic right ventricular cardiomyopathy (ARVC), and restrictive cardiomyopathy (RCM) [1]. Among these, DCM is a leading cause of heart failure (HF) and the primary cause of heart transplantation [2]. This condition is characterized by ventricular dilation and impaired systolic function of one or both ventricles, in the absence of significant loading conditions [1]. Genetic mutations account for approximately 35% of DCM cases [2, 3]. Among these, mutations in structural proteins, such as filamins, are increasingly recognized as significant contributors [3, 4].

Filamins are a family of cytoskeletal proteins that anchor various transmembrane proteins to the actin cytoskeleton [5]. Among them, abnormalities of Filamin C, secondary to mutations on the *FLNC* gene, are associated with cardiac disorders that result in diverse cardiomyopathy phenotypes. These mutations are linked to a malignant clinical course, characterized by a high risk of arrhythmias and sudden cardiac death (SCD) [6–8]. Therefore, early detection and management are crucial to preventing adverse outcomes in these patients. We present the case of a young patient who suffered an ischemic stroke, later diagnosed with genetically determined DCM caused by a single mutation in the *FLNC* gene.

## 2. Case Presentation

A 28-year-old male with no significant cardiovascular history but with a family history of HF in his maternal grandfather, presented to the emergency department with symptoms of an ischemic stroke, including aphasia, facial paralysis, right hemiparesis, and sixth cranial nerve involvement. His initial National Institutes of Health Stroke Scale (NIHSS) score was 13, indicating a moderate neurological deficit. Thrombolysis led to significant neurological improvement, with his NIHSS score decreasing to 4. A post-thrombolysis cranial computed tomography revealed a left frontal cortical-subcortical hypodensity.

The patient's young age and the unexpected stroke prompted an extensive evaluation for secondary causes. Infectious panels for hepatitis, HIV, and syphilis, as well as thrombophilia and rheumatologic studies, returned negative results. The 24-h Holter monitoring showed no atrial fibrillation (AF) linked to the ischemic event. Additionally, no ventricular ectopic beats were detected. Transesophageal echocardiography (TEE) was then performed to identify potential embolic sources. While no embolic source was detected, TEE revealed severe left ventricular systolic dysfunction with a left ventricular ejection fraction (LVEF) of 20%. At discharge, the patient was started on a combination of medications, including a beta-blocker (BB) (bisoprolol, 2.5 mg once daily), a sodium–glucose cotransporter 2 (SGLT2) inhibitor (empagliflozin, 10 mg once daily), an angiotensin-converting enzyme (ACE) inhibitor (enalapril, 2.5 mg every 12 h), a heart rate–reducing agent (ivabradine, 5 mg once daily), a statin (atorvastatin, 80 mg once daily), and a loop diuretic (furosemide, 20 mg every 12 h)

**Figure 1:**
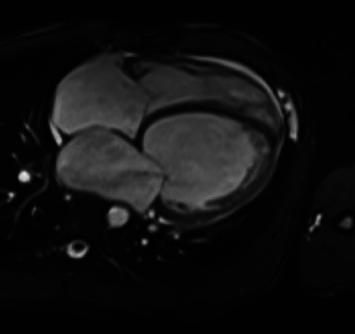
Cardiac magnetic resonance imaging showing a four-chamber view with evidence of biventricular dilatation.

After discharge, the patient experienced two episodes of decompensation, each presenting as acute pulmonary edema. Cardiac magnetic resonance imaging (MRI) confirmed severe biventricular dilated cardiomyopathy, characterized by significant LVEF 15% as well as right ventricular dysfunction, with a right ventricular ejection fraction of 16%. Additional findings included left atrial enlargement and moderate mitral regurgitation. There was no evidence of myocardial edema, fibrosis, or inflammatory or infiltrative processes (Figure 1).

At his first follow-up, the patient was classified as New York Heart Association class II. Due to the potential risk of occult AF, warfarin was added to his regimen, administered as 10 mg and 5 mg on alternating days. A subsequent electrocardiogram revealed a left bundle branch block (LBBB) with a QRS duration of 200 milliseconds. With no definitive cause identified, genetic testing was pursued to investigate possible DCM-related genetic mutations and Fabry disease. The testing identified a heterozygous variant in the *FLNC* gene: NM_001458.5(FLNC):c.1156G>T; p.Glu386∗ (Table 1).

At the second follow-up, the patient's ACE inhibitor was replaced with an angiotensin receptor–neprilysin inhibitor (sacubitril-valsartan, 50 mg every 12 h). Given the increased risk of SCD, he was referred for primary prevention with an implantable cardioverter-defibrillator (ICD). Genetic testing was also performed on his first-degree relatives.

## 3. Discussion

We identified a genetically determined case of DCM caused by a single *FLNC* gene mutation in our patient. While HCM is the cardiomyopathy most frequently linked to genetic causes, mutations account for up to 35% of DCM cases [2]. The *FLNC* gene encodes Filamin C, a dimeric structural protein with an actin-binding domain, predominantly expressed in striated muscles, including cardiac and skeletal muscle. Filamin C is localized to the Z-disc, sarcolemma, myotendinous junctions, and intercalated discs, where it maintains sarcomere integrity by crosslinking actin filaments and anchoring sarcolemmal proteins to the cytoskeleton [9]. *FLNC* mutations are associated with a spectrum of cardiac phenotypes and operate through three mechanisms: (1) misfolded proteins overwhelming proteasome and autophagy pathways; (2) toxic gain-of-function effects altering ligand-binding properties; and (3) premature termination codons causing nonsense-mediated decay and haploinsufficiency [10]. While mechanisms (1) and (2) refer to nontruncating (missense) mutations, commonly associated with HCM, mechanism three involves truncating (nonsense) mutations, primarily linked to DCM and, more specifically, to arrhythmogenic cardiomyopathy [7, 11–13].

Although robust data on DCM prevalence is limited, it is estimated to be approximately twice as common as HCM, which has a prevalence of one in 250–500 adults [14]. Among these, the prevalence of *FLNC* variants is low, affecting only 3% of cases [15]. Truncating mutations are even rarer, accounting for only 3%-4% of *FLNC* variants [16]. Despite its low prevalence, DCM caused by truncating mutations in the *FLNC* gene often follows a more severe course, with higher risks of major ventricular arrhythmias (MVA), myocardial fibrosis, and SCD [1, 7, 8, 17]. These variants have been associated with a penetrance exceeding 97% in carriers older than 40 years [7]. The early and severe cardiac involvement observed in our 28-year-old patient underscores the potential for aggressive phenotypic expression even in younger individuals. Considering potential genetic causes in DCM diagnosis and management is crucial, as identifying variants like the one in our case could impact prognosis and guide treatment.

The mean age of onset is typically in early-to-mid adulthood [16]. However, most patients begin to exhibit symptoms around 40 years of age. For instance, a study demonstrated that the median age for patients with *FLNC*-related DCM was 40 years, with over 60% of these patients being older than 35 years [18]. Consequently, the early presentation in our patient is atypical. On a regular basis, clinical presentation includes classical HF symptoms [1]. The presence of a related ischemic stroke could be explained by a greater thromboembolic risk due to an increased risk of AF [19]. However, it is more associated with RCM and HCM than DCM what could explain its absence of electrocardiographic monitoring. Nevertheless, the presentation of a related stroke makes our case distinctive. These patients also experience complex ventricular arrhythmias. A pooled analysis of patients with filamin-related cardiomyopathy demonstrated a high rate of ventricular ectopy on 24-h Holter monitoring; however, this was not observed in our patient, highlighting the phenotypic variability of *FLNC* truncating variants [18].

*FLNC*-associated DCM predominantly affects the left ventricle without involving the right ventricle. This observation is particularly significant as a LVEF below 35% has been proposed as a risk factor for SCD [1]. However, evidence from other studies does not consistently support a strong association between these factors [18]. Notably, our patient showed global ventricular deterioration, which is atypical for *FLNC* related DCM. We hypothesize that the right-sided dysfunction may result from long-standing left-sided disease, given the unclear duration of disease progression. Alternatively, this could reflect the overlap between cardiomyopathy phenotypes associated with *FLNC* variants, which have been linked to HCM, NDLVC, RCM, and, particularly, ARVC, due to its association with right ventricular dilation [1, 12, 16, 20]. Moreover, the relationship between our patient's specific genetic mutation—associated with hypertrophic and restrictive cardiomyopathy, as shown in the cardiomyopathy panel—and the imaging findings of ventricular dilatation supports the concept of overlapping phenotypes.

Electrocardiographic findings often include low QRS voltages and negative T waves and lateral leads, with the latter associated with higher risk of SCD [18]. In our patient, we observed a LBBB lasting 200 milliseconds. While LBBB has been reported in cases of *FLNC*-associated cardiomyopathy, it is more commonly associated with ARVC, further supporting the overlap between these cardiac phenotypes [1, 15]. Although not observed in our patient, 75% of patients exhibit late gadolinium enhancement on cardiac MRI, a significant marker of disease severity, since literature consistently correlates fibrosis on cardiac MRI with a higher risk of SCD, MVA and end-stage HF [18, 21, 22].

As previously discussed, the risk of SCD is a central concern in the clinical management of DCM. The Filamin C Registry Consortium recently proposed a five-variable model for predicting SCD or MVA in carriers of *FLNC* truncating variants, including age, male sex, history of syncope, nonsustained ventricular tachycardia, and LVEF. Although the relationship between LVEF and arrhythmic risk is nonlinear, values above 58% are associated with significantly lower risk [23]. In our patient, the presence of high-risk features such as male sex and severely reduced LVEF highlights the importance of systematic risk assessment and supports the need for vigilant longitudinal monitoring and individualized risk stratification, even in the absence of overt electrical abnormalities at baseline.

Management includes standard HF treatment with renin–angiotensin–aldosterone system inhibitors, BBs, SGLT2 inhibitors, and diuretics, following clinical practice recommendations. Anticoagulation therapy is individualized and advised in patients with DCM who are at risk for thromboembolism, such as those with AF. In such cases, direct oral anticoagulants are generally preferred over vitamin K antagonists. Current guidelines also recommend cardiac MRI for SCD risk stratification and ICD implantation as a primary preventive measure in patients with high-risk features, such as significant myocardial fibrosis or LVEF ≤ 35%, as observed in our patient. Genetic testing is strongly advised for patients with DCM, as it can confirm the diagnosis, assess prognosis, and guide treatment. Following the identification of a likely pathogenic *FLNC* truncating variant, genetic testing was also pursued in first-degree relatives to explore potential familial expression. For instance, the reported history of HF in the patient's maternal grandfather may represent an unrecognized familial manifestation, further supporting cascade screening as recommended by current guidelines [1].

## 4. Conclusion

This case highlights the importance of identifying genetic causes, particularly *FLNC* mutations, in young patients with unexplained cardiomyopathy. Although *FLNC*-associated DCM is rare, its link to major arrhythmias and SCD underscores the need for early genetic testing and comprehensive management. Our patient's atypical presentation suggests an overlap of phenotypes, which should be considered in patient management. HF standard treatment, SCD stratification through cardiac MRI, proactive ICD implantation, and genetic testing are crucial for managing the condition and improving outcomes.

## Figures and Tables

**Table 1:** Cardiomyopathy panel with a positive result for a pathogenic *FLNC* variant.

Gene	Chromosome
*ACTC1*	15q14
*FLNC*	**7q32.1**
*LAMP2*	xq24
*MYL2*	12q24q.11
*PRKAG2*	7q36.1
*TNNI3*	19q13.42
*TTR*	18q12.1
*CSRP3*	11p15.1
*GLA*	xq22.1
*MYBPC3*	11p11.2
*MYL3*	3p21.31
*PTPN11*	12q24.13
*TNNT2*	1q32.1
*DES*	2q35
*JPH2*	20q13.12
*MYH7*	14q11.2
*PLN*	6q22.31
*TNNC1*	3p21.1
*TPM1*	15q22.2

*Note:* Result: Presence of the variant described as NM_001458.5(FLNC):c.1156G>T; p.Glu386∗, classified as likely pathogenic, in heterozygosity in the *FLNC* gene. This variant is associated with familial hypertrophic cardiomyopathy 26 (OMIM 617047) and restrictive cardiomyopathy 5 (OMIM 617047) with autosomal dominant inheritance. Conclusion: Presence of a likely pathogenic heterozygous variant in the *FLNC* gene. Additional variant details: Genomic position: chr7: 128, 478, 429 (GRCh37/hg19). Methodology: Target‐capture next‐generation sequencing of exonic regions. Coverage: > 20× in 100% of targeted bases. Zygosity/VAF: Heterozygous (∼50% variant allele frequency). The chromosomal location of the affected gene is highlighted in bold.

## Data Availability

Data sharing is not applicable to this article as no datasets were generated or analyzed during the current study.
